# Earthquake Nucleation Size: Evidence of Loading Rate Dependence in Laboratory Faults

**DOI:** 10.1029/2018JB016803

**Published:** 2019-01-22

**Authors:** Simon Guérin‐Marthe, Stefan Nielsen, Robert Bird, Stefano Giani, Giulio Di Toro

**Affiliations:** ^1^ Department of Earth Sciences Durham University Durham UK; ^2^ Department of Engineering Durham University Durham UK; ^3^ Department of Geosciences University of Padova Padova Italy

**Keywords:** nucleation length of earthquakes, seismicity patterns of subduction zones, laboratory fault

## Abstract

Recent Global Positioning System observations of major earthquakes such as the 2014 Chile megathrust show a slow preslip phase releasing a significant portion of the total moment (Ruiz et al., 2014, https://doi.org/10.1126/science.1256074). Despite advances from theoretical stability analysis (Rubin & Ampuero, 2005, https://doi.org/10.1029/2005JB003686; Ruina, 1983, https://doi.org/10.1029/jb088ib12p10359) and modeling (Kaneko et al., 2017, https://doi.org/10.1002/2016GL071569), it is not fully understood what controls the prevalence and the amount of slip in the nucleation process. Here we present laboratory observations of slow slip preceding dynamic rupture, where we observe a dependence of nucleation size and position on the loading rate (laboratory equivalent of tectonic loading rate). The setup is composed of two polycarbonate plates under direct shear with a 30‐cm long slip interface. The results of our laboratory experiments are in agreement with the preslip model outlined by Ellsworth and Beroza (1995, https://doi.org/10.1126/science.268.5212.851) and observed in laboratory experiments (Latour et al., 2013, https://doi.org/10.1002/grl.50974; Nielsen et al., 2010, https://doi.org/10.1111/j.1365-246x.2009.04444.x; Ohnaka & Kuwahara, 1990, https://doi.org/10.1016/0040-1951(90)90138-X), which show a slow slip followed by an acceleration up to dynamic rupture velocity. However, further complexity arises from the effect of (1) rate of shear loading and (2) inhomogeneities on the fault surface. In particular, we show that when the loading rate is increased from 10^−2^ to 6 MPa/s, the nucleation length can shrink by a factor of 3, and the rupture nucleates consistently on higher shear stress areas. The nucleation lengths measured fall within the range of the theoretical limits L_b_ and $L_{\infty }$ derived by Rubin and Ampuero (2005, https://doi.org/10.1029/2005JB003686) for rate‐and‐state friction laws.

## Key Points

- The nucleation length decreases with loading rate, implying that smaller‐size asperities clusters can be triggered by accelerated slip
- The nucleation position localizes on high coulomb stress patches with small‐scale inhomogeneities at high loading rates
- The measured nucleation length of laboratory earthquakes falls into the range predicted by numerical and theoretical studies

## 1 Introduction

The precursory phase of earthquakes and, more generally, the different phases of the seismic cycle remain in large part poorly understood. However, some promising advances have been made in the past decades thanks to fault observations, theoretical and numerical models, and small‐scale laboratory experiments.

It is well‐known that some faults are able to release a significant portion of the strain energy accumulated during the tectonic loading phase by slow, aseismic creep (Kanamori, 1977; Scholz et al., 1969). The observation of small to moderate earthquakes and repeaters evolving in the area of an impending earthquake has allowed to infer either the presence of slow slip (Hasegawa & Yoshida, 2015; A. Kato et al., 2012) or the advancement of a slow rupture front (Bouchon et al., 2011; Bouchon et al., 2013). More recently, thanks to substantial developments of GPS and cGPS networks along with satellite interferometry, it has been possible to use geodetical data in conjunction with seismic signals to highlight the crustal deformation during different stages of the seismic cycle. In a small number of cases so far, an accelerated slip phase preceding large or great earthquakes of several weeks (Ruiz et al., 2014, 2017) to months (Socquet et al., 2017) has been identified, often accompanied by seismic swarms triggered as the slip progresses (A. Kato et al., 2012). The latter may be indicative of the nucleation process and has been interpreted as part of the triggering mechanism of earthquakes (Ruiz et al., 2014, 2017). In the later case, the coseismic slip area was smaller and located inside the large nucleation zone that started slipping a few months before. More recently, Tape et al. (2018) showed that a M3.7 earthquake in Alaska initiated with the acceleration of a rupture front 22 s before the main shock. This last observation concerns a small earthquake rupture and thus provides insight in the rupture process at an intermediate scale between laboratory experiments and great earthquakes.

While the above observations are provoking, they are so far too few to clearly quantify the prevalence of preslip and to demonstrate a statistically significant causality relation between slow‐slip and earthquake nucleation. As a consequence, opposing models have been proposed for earthquake initiation: the preslip model of Ellsworth and Beroza (1995) and the cascade model (Olson & Allen, 2005). The main difference between the two models is that in the preslip model, the rupture expands until the slipping patch reaches a critical size *L*_*c*_ at which it becomes unstable. In the cascade model, small and large earthquakes start in a similar manner, by successive random breaking of asperities eventually leading to a large rupture for which the size cannot be predicted until it stops (Olson & Allen, 2005).

As it has been suggested that a number of large events have been triggered by preslip (Ruiz et al., 2014, 2017; Tape et al., 2018), it is crucial to understand how large can a slipping patch grow before being likely to become unstable and trigger a major event, and what controls the size of L_c_, intended as the size above which a sliding fault patch will start to propagate spontaneously. L_c_ can be predicted for a few simple models related to earthquake faulting. Assuming that the stress drop inside the slipping patch is known, an estimate of L_c_ can be obtained based on energy concepts, stemming from the original Griffith criterion for brittle failure (Griffith, 1921), subsequently extended to elasto‐plastic materials (Irwin, 1957; Rice, 1968) and to shear rupture on frictional earthquake faults (Andrews, 1976). The resulting L_c_ decreases with the normal stress and the stress drop within the nucleation patch, and increases with the fracture energy. For a homogeneous fault characterized by a velocity‐weakening friction law, the size L_b − a_ of an unstable slipping patch can be predicted (Gu et al., 1984; Ruina, 1983) by stability analysis. Some correspondence between L_b − a_ (obtained in terms of stability) and L_c_ (obtained in terms of energy balance) must exist and has been explored for particular cases of rate‐and‐state friction laws (Rubin & Ampuero, 2005; Uenishi & Rice, 2003). Importantly, the study of Rubin and Ampuero (2005) using rate‐and‐state models shows that L_b − a_ is not unique but that there exists a range of possible nucleation lengths within the values $\left(L_{b},L_{\infty }\right)$, corresponding to the lower and upper bounds of the nucleation length predicted, respectively (see section 4). While these theoretical predictions are roughly matched by experimental observations, a more complex behavior is observed on experimental and natural faults, possibly due to strong inhomogeneity (Harbord et al., 2017).

Because of the small number of preslip observations in nature and the complexity of the physical processes involved at different scales, one way to investigate the dependence of *L*_*c*_ on individual physical parameters is to replicate this slow slip in controlled laboratory experiments (N. Kato et al., 1992; Latour et al., 2013; Mclaskey & Yamashita, 2017; Ohnaka & Kuwahara, 1990; Ohnaka, 1992; Xu et al., 2017) and in numerical models (N. Kato & Hirasawa, 1996; Kaneko & Ampuero, 2011; Kaneko et al., 2016; Kaneko et al., 2017; Rubin & Ampuero, 2005). Latour et al. (2013) evidenced that *L*_*c*_ was inversely proportional to the normal stress using polycarbonate plate as earthquake laboratory analog. The normal stress dependence is supported by theoretical studies using rate‐and‐state or slip weakening friction laws and crack stability analysis Ruina (1983). Ohnaka (1992) and N. Kato et al. (1992) also observed a similar pre‐slip in laboratory rupture experiments using granite slabs. The scaling of *L*_*c*_ in those experiments depends on normal stress, fault surface roughness and slip weakening distance *D*_*c*_, which is the amount of relative slip on a fault needed for the friction to reach the dynamic value. The effect of normal stress is also evidenced in numerical models (Kaneko et al., 2016). But, more than the *L*_*c*_ dependence on normal stress, numerical models also show that increasing the loading rate causes *L*_*c*_ to decrease, using rate‐and‐state friction laws (N. Kato & Hirasawa, 1996; Kaneko et al., 2016).

In order to verify this decrease of L_c_ , we conducted experiments similar to the ones of Latour et al. (2013), but this time investigating the effect of the loading rate on the nucleation length of laboratory ruptures. Even though experiments using granite blocks have already investigated the L_c_ dependence on loading rate, this was done either by looking at strain gages signals (N. Kato et al., 1992) or by observing the transition between stable and unstable behavior of granite slabs of length close to L_c_ (Mclaskey & Yamashita, 2017). The advantage of the photoelastic technique used here is that the tips of the propagating rupture can be directly tracked, allowing to measure nucleation length and position.

## 2 Materials and Methods

**Figure 1:**
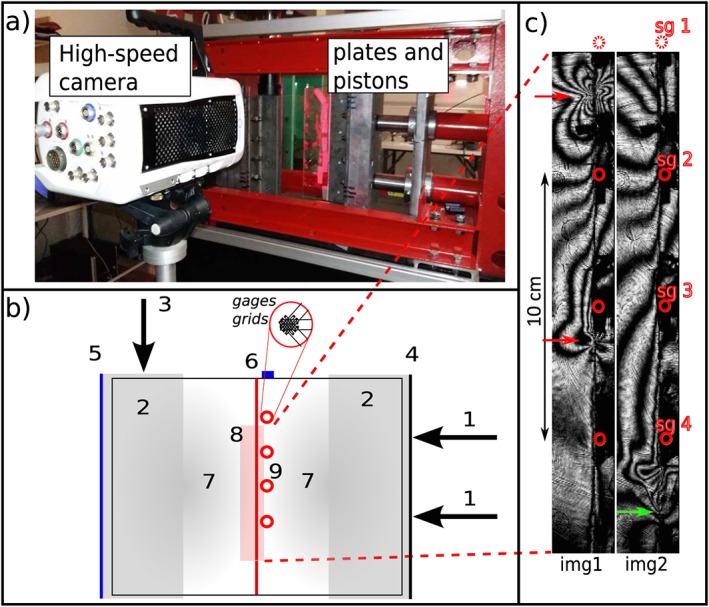
Photograph and schematic description of the experimental setup. (a) Picture of the biaxial press used for the rupture experiments. (b) Sketch of the loading and monitoring configuration with the two polycarbonate plates (7) held through metallic clamps (2). Two horizontal pistons (1) apply a distributed normal force through the right‐hand clamp edge (4), which is fixed in the vertical direction but allowed to move in the horizontal direction. A vertical piston (3) actuates the shear stress by applying a vertical force through the left‐hand clamp edge (5), which is allowed to move vertically along a low friction rail, but is fixed in the horizontal direction. Four strain gage rosettes (9) indicated by red circles are fixed using cyanoacrylate bond along the 30 × 1 cm interface (red line) between the two plates. An accelerometer (6) is fixed at the top of the right plate, and a high‐speed camera captures the rupture events in a 20‐cm‐long window (8) in the middle of the interface. (c) Two examples of isochromatic fringe patterns recorded during a rupture. The slipping zone of img1 is situated between the two red arrows that represent the crack tips, or propagating rupture fronts. The green arrow on img2 points at the bottom tip that has transitioned to supershear velocity, while the top tip has propagated outside of the camera field. Again, red circles represent the strain gages, which are numbered sg1–4.

The laboratory setup (Figures 1a and 1b) consists in two rectangular polycarbonate plates 30 × 15 × 1 cm, held in sliding contact across their 30‐cm edge.

Two horizontal pistons apply a uniform pressure through a metal holder, maintaining the 30 cm by 1 cm contact interface under normal stress. A third piston applies a vertical force on the metal holder of the left‐hand plate, thus imposing shear stress on the sliding interface in a simple shear configuration. All pistons are controlled by hand pumps. The two horizontal pistons and the normal stress remain fixed during the shear loading phase leading to the rupture. After each rupture the normal stress is released, the plates are reset to their initial positions, and the normal stress is imposed again for the next experiment. Three strain components are monitored at each of the four strain gage rosette locations (2 mm away from the fault interface and equally spaced 5 cm away from each other).

**Figure 2:**
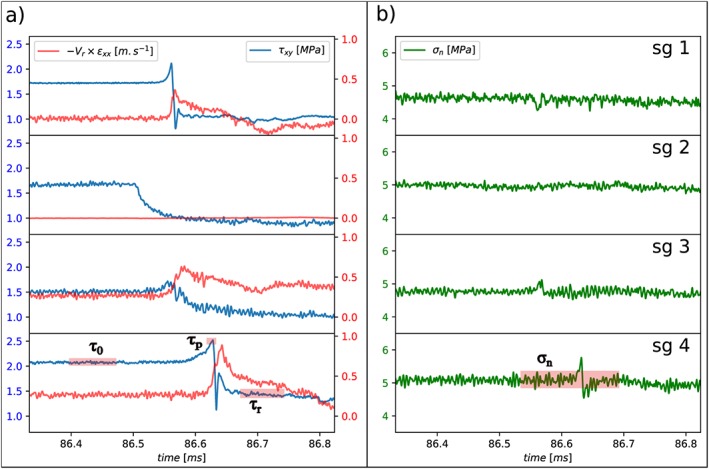
Example of strain gage signals recorded during a rupture that initiates close to the strain gage sg 2. (a) Shear stresses τ_xy_ and fault parallel particle velocities −V_r_ ε_xx_ recorded at the four different locations corresponding to sg1 to sg4, V_r_ being the rupture velocity . (b) Normal stresses σ_n_ recorded at the same locations. For one event, the initial shear stressesτ_0_ , peak stresses τ_p_ , residual stresses τ_r_, and normal stressesσ_n_ are picked as averaged values in time windows shown on the stress records at sg4. Note that although σ_n_ can slightly vary locally at the rupture front, we only pick the averaged value.

The signals are sampled at 10 MHz and filtered at 500 kHz in order to record the initial, peak, residual, and normal stresses before the rupture, respectively, τ_0_,τ_p_,τ_r_, and σ_n_ (see Figure 2). Because we do not control the exact value of shear loading rate $\dot{\tau }$ when moving the vertical piston using the hand pump, we also use the strain gages to measure the average loading rate $\dot{\tau }=\Delta \tau /\Delta t$ during the few seconds of the loading phase.

**Figure 3:**
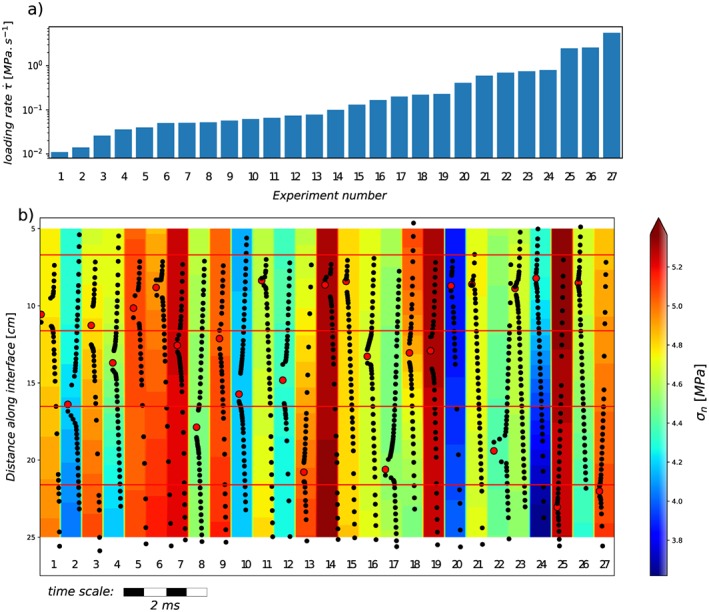
(a) Loading rate for each experiment and (b) corresponding rupture histories presented in individual time windows of 500 μs. The black dots are the positions of the crack tips. The background colors represent the normal stress distribution for each rupture, measured with the four strain gage rosettes, and interpolated between their positions indicated by the red solid lines. The big red dots are the nucleation positions.

**Figure A1:**
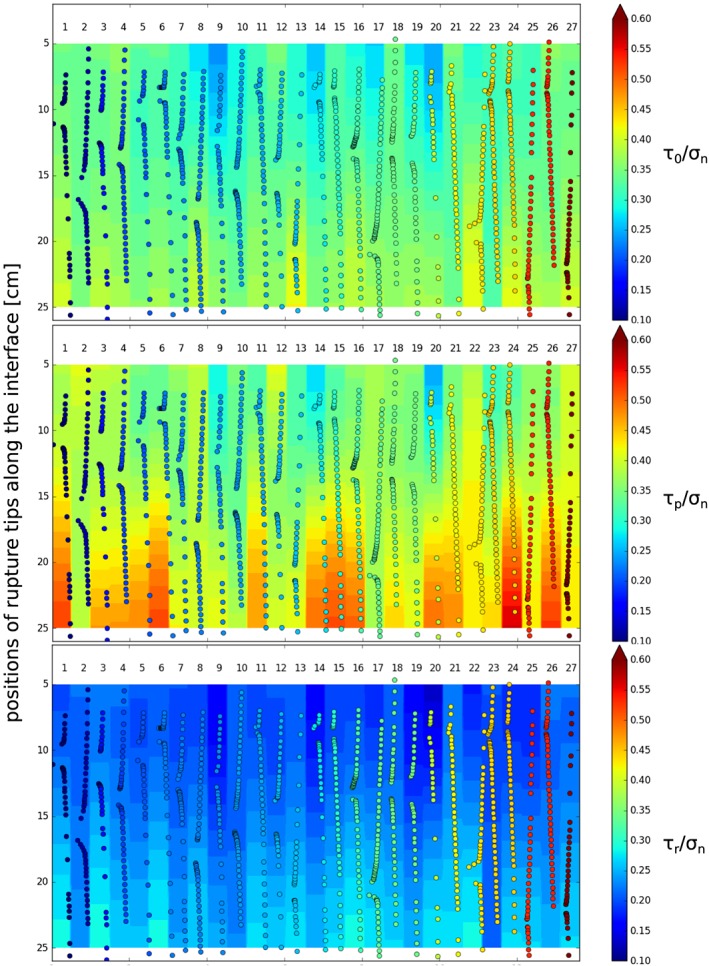
Maps for each 27 experiments of the friction before, during and after the passage of the rupture front. Friction coefficients are extrapolated between the four strain gages locations, using values picked as in Figure 2. The dot colors represent the loading rate and follow the same scale than on Figure 4a.

A high‐speed camera continuously overwrites a circular buffer, recording at 210^5^ frames per second. A signal is sent to the camera immediately after the dynamic rupture, allowing to store the frames from the last few seconds before rupture, and up to approximately 0.5 s after. We use the well‐known photoelastic properties of the polycarbonate to visualize so‐called isochromatic fringes, highlighting the areas of high stress concentration that correspond to the edges of the slipping patch (Figure 1c), as used in previous laboratory rupture experiments (Nielsen et al., 2010; Rosakis et al., 2007). We select the frames where sharp stress variations start to appear along the contact interface (start of nucleation), until the crack propagates dynamically and the tips reach the limit of the camera field. Thus, for each rupture experiment, which lasts a few hundreds of microseconds, we are able to track the positions of the rupture tips versus time along with the absolute values of stress measured at four locations thanks to the strain gage rosettes. The gages' values can be interpolated to obtain a continuous stress distribution (see Figures 3b and A1). The two surfaces in contact were initially smoothed using 400 grit diamond powder and then sandblasted with heterogeneous sand particles to simulate a self‐similar roughness in a similar way than in Lu et al. (2010). Note that before the experiments presented below, several stick‐slip events had already been triggered. This might have introduced defects on the simulated fault surface and would explain why small‐scale stress heterogeneities (wavelengths of 4 to 8 mm) are visible in Figure 7. Those short wavelengths are superimposed to larger normal and shear stress variations that are explained by the loading conditions (see Appendix D).

## 3 Results

We conducted a total of 27 individual experiments, at imposed loading rates ranging from 0.01 to 6 MPa/s (Figure 3a), with normal stresses maintained around 4.7 ± 0.8 MPa (Figure 3b). For each experiment, we present the tracking of the rupture tip positions in individual time windows representing 500 μs. Crack tips are not clearly visible on all frames (due to weak stress concentration or to masking by strain gage rosettes), which is why there are apparent gaps in picking of the front positions (black dots) in Figure 3b. We took care of performing the experiments in a random order in terms of the background loading rate, to exclude any possible bias due to progressive sample wear. As a consequence the rupture histories presented in Figure 3b have not been run in the same order as presented (by increasing loading rate). The background color represents the normal stress distribution for each individual experiment, interpolated from the four strain gage rosettes values.

**Figure 4:**
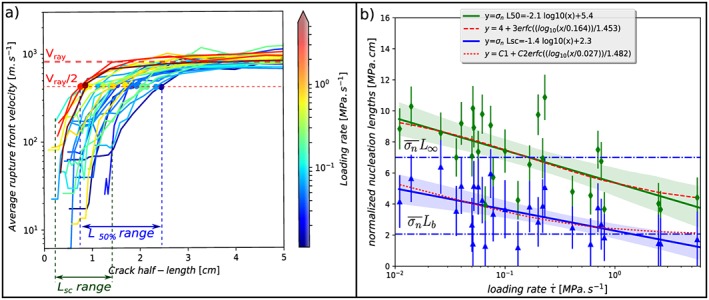
(a) Characteristics of the nucleation phase of each individual rupture experiment under different background loading rates. Warmer colors represent higher loading rates. The dots represent a nucleation length taken arbitrarily as the crack half‐length when the average rupture front velocity $\bar{V_{r}}$ reaches 50% of the Rayleigh wave speed V_Ray_. (b) Normalized nucleation lengths L_sc_ (blue triangles) at the start of the acceleration and L_50%_ (green diamonds) by the normal stress, as a function of the background loading rate $\dot{\tau }$. The blue and green areas represent the uncertainty on the arbitrary linear relations plotted as a solid lines, using a bootstrap method at 95.45% of confidence (2 × STD), for $\bar{\sigma_{n}}L_{\mathrm{sc}}$ vs $\dot{\tau }$ and $\bar{\sigma_{n}}$ L_50%_ versus $\dot{\tau }$, respectively. The dashed red lines are nonlinear fits assuming that the nucleation length tends asymptotically toward limiting values L_b_ and $L_{\infty }$ at high and low values of $\dot{\tau }$, respectively.

Using the position of the rupture tips versus time, we can plot the average rupture front velocity $\bar{V_{r}}$ versus crack half‐length (Figure 4a; $\bar{V_{r}}$ being the average of the two rupture fronts velocities in the case of bilateral ruptures). We see clearly the phase of acceleration of *V*_*r*_ up to about the Rayleigh wave speed *V*_*Ray*_≈820m/s, that is, the limiting velocity for subshear ruptures (dashed red line in Figure 4). We use *V*_*Ray*_ to define *L*_50%_ as the crack half‐length when $\bar{V_{r}}=0.5\;V_{\mathrm{Ray}}$. We also observe supershear rupture in some of the experiments (Figure 1c) in particular when the length of the propagating rupture exceeds 5 cm (not shown in Figure 4a). (The limiting rupture velocity in supershear is the *P* wave velocity ≈ 1,860 m/s, but we always use *V*_*Ray*_ for the definitin of *L*_50%_). Using *L*_50%_, the stress concentration at the crack tips is large enough to allow a clearly visible rupture tips in most cases. For comparison to the theoretical predictions *L*_*b*_,*L*_*b* − *a*_ and $L_{\infty }$, we also define *L*_*sc*_ as the nucleation length at which nucleation starts to accelerate. However, at such early stage of nucleation, the crack tip position is less clear; therefore, *L*_*sc*_ is poorly resolved, in particular for small nucleation sizes (see lower bound of *L*_*sc*_ range; Figure 4a).

We observe a shrinking of the nucleation length with increasing loading rate in accordance to the numerical model of Kaneko et al. (2016). *L*_50%_ shrinks from almost 2.5 cm to approximately 0.8 cm when the loading rate is increased from 10^−2^ to 6 MPa/s respectively. Although the variability is high, this shows clearly that *L*_50%_ is dependent on the loading rate.

Because the nucleation length is also inversely proportional to the normal stress (see section 4), we normalize *L*_50%_ by multiplying it by *σ*_*n*_. When plotted versus $\dot{\tau }$ the normalized nucleation length decreases with increasing loading rate (Figure 4b). As we have an uncertainty on the nucleation length and on the normal stress interpolated at the nucleation position, we propagate the uncertainty on *L*_50%_ ×*σ*_*n*_ and plot the error bars. Even though we do not know the exact relationship between normalized *L*_50%_ and loading rate, a linear regression shows a clear negative slope:

(1) $$L_{50\%}\times \sigma_{n}=-2.1\log_{10}\dot{\tau }+5.4.$$

*L*_50%_ is in cm, *σ*_*n*_ in MPa, and $\dot{\tau }$ in MPa/s. Dividing equation (1) by the average normal stress of $\bar{\sigma_{n}}$ = 4.7 MPa, we obtain

(2) $$L_{50\%}=-0.44\log_{10}\dot{\tau }+1.15.$$

The uncertainty on this slope is calculated using a bootstrap method and displayed as a light green area. The regression coefficient is clearly negative meaning that the normalized nucleation length is dependent on the loading rate.

Assuming that the nucleation length *L*_*sc*_ tends asymptotically toward the theoretical limiting values *L*_*b*_ and $L_{\infty }$ at high and low values of $\dot{\tau }$, respectively (see section 4), we can also obtain an empirical fit using the following mathematical form (using erfc to produce tapering at both high and low values of $\dot{\tau }$):

(3) $$\bar{\sigma_{n}}L_{\mathrm{sc}}=c1+c2\times \text{erfc}\left(\frac{\log_{10}(\dot{\tau }/c3)}{c4}\right),$$

where $c1=\bar{\sigma_{n}}L_{b}=2.07\;[\text{MPa.cm}],c2=\bar{\sigma_{n}}(L_{\infty }-L_{b})/2=2.47\;[\text{MPa.cm}],c3=0.027\;[{\text{MPa.s}}^{-1}]$, and *c*4 = 1.482.

A similar relation can be found using *L*_50%_ (Figure 4b).

**Figure 5:**
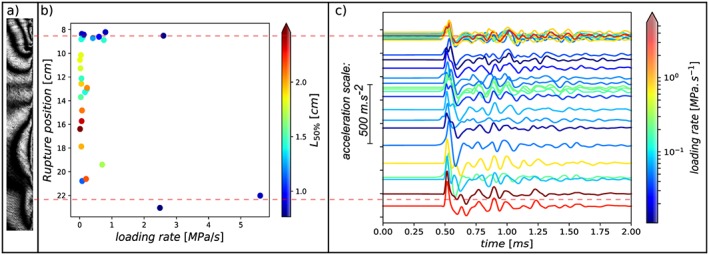
(a) Picture of the typical isochromatic fringe pattern taken approximately 500 μs before nucleation, highlighting high shear and normal stresses areas around 8.5 and 22.5 cm. (b) Nucleation location of each individual experiment. The nucleation location is taken as the mean value of the first picked positions of the crack tips propagating upward and downward along the interface. The dots color represents the nucleation length L_50%_ measured as in Figure 4a. (c) Accelerations recorded at the top edge of the right plate (see Figure 1, 2). The initial accelerations are zeros, and an offset is applied to plot the waveforms at the position where the rupture has nucleated.

In addition to the shrinking of *L*_*c*_ with increasing loading rate, we observe that the nucleation position along the interface is not random but also affected by $\dot{\tau }$. Indeed, at loading rates over 0.3 MPa/s the nucleation localizes only on areas situated around 8.5 and 22.5 cm along the interface. The nucleation length *L*_*c*_ is small every time the rupture nucleates on those patches (Figure 5b), and the accelerations (recorded at the top of the right plate) show that all the ruptures initiating around 8.5 cm have very similar waveforms (Figure 5c). We find that the preferred nucleation sites at high loading rates correspond to areas of relatively higher shear and normal initial stresses by using the typical photoelastic fringes pattern before nucleation (taken approximately 500 μs before the crack tips become visible, as shown in Figure 5a) and the strain gages data; the method is detailed in Appendix D. Although the exact stress distribution is different between experiments (see Appendix A), the general fringe pattern before rupture is consistent and always showing the two same high‐stress areas. The stress variations from one experiments to another are of too short wavelength or too small magnitude to be quantified using the method described in Appendix D. The sparse strain gage measurements do not enable to resolve sharp stress heterogeneities either. At low loading rates, the nucleation is observed to start in more homogeneous zones (between 10 and 18 cm), with apparent slightly lower initial stress values.

**Figure 6:**
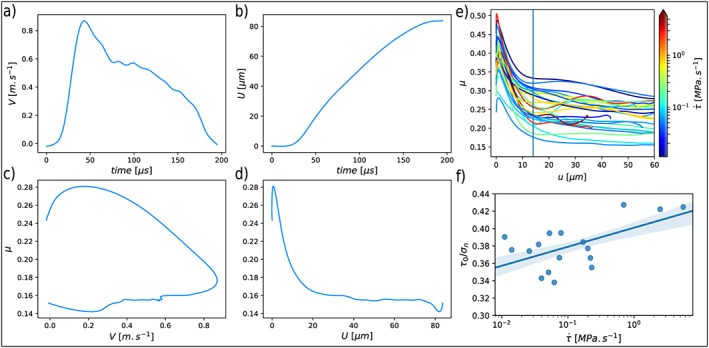
a) Plot of the local slip velocity V=−2V_r_ ε_xx_ (ε_xx_ is the fault parallel strain low‐pass filtered at 30 kHz) used to calculate (b) the relative displacement U, as detailed in Appendix C. The friction evolution is then plotted as a function of (c) the local slip velocity V and (d) the relative displacement U. e) Dynamic slip‐weakening friction laws of each experiments. f) Normalized τ_0_ vs loading rate for all events excepts the ones having nucleated around 8.5 cm along the interface which are discarded as the stress values are not resolved by the sparse strain gage measurements.

Finally, we attempt to derive the dynamic slip‐weakening friction laws of each rupture (Figure 6e) using the method described in Appendix C. From the camera data we select strain gages that recorded *ε*_*xx*_ when the rupture front attained a quasi‐static propagation velocity *V*_*r*_≈*V*_*Ray*_ and use the measured velocity to calculate *u*. Filtering the signal at 30 kHz and plotting the friction evolution versus slip for each experiment indicates a slip dependence of friction with a consistent critical slip weakening distance *D*_*c*_≈ 14 μm (Figure 6e). However, there is no clear trend concerning the slip weakening dependence of friction with regard to the loading rate. This should be expected as the dynamic friction is weakly related to the quasi‐static friction law state variables and therefore to the loading history. To quantify what happens during the quasi‐static stage of rupture, we use the values of *τ*_0_ and *σ*_*n*_ interpolated at the nucleation position (Appendix A) and plot their relation with $\dot{\tau }$ (Figure 6f). The events nucleating on the high‐stress area around 8.5 cm along the interface are discarded, as the interpolated stress distribution using the strain gages can clearly not resolve the stress field visible on the isochromatics (Figure 5a). The ratio *τ*_0_ / *σ*_*n*_ seems to increase with loading rate, which is consistent with the rate effect of rate‐and‐state friction laws. A possible microphysical interpretation is that locked microcontacts across the sliding surface deform plastically under shear; in that case their shear stress is expected to increase with strain rate. We can also infer that if the higher stresses around 8.5 cm would be properly resolved by the strain gages, this would add more point in the upper right part of the graph Figure 6f.

## 4 Discussion

We now discuss our observations on the size of experimental nucleation to the theoretical predictions that can be made using stability analysis and assuming some type of rate‐and‐state frictional behavior.

Because the stability analysis does not consider a change in the remote load, it is expected that those experiments where the loading rate is the smallest should be closer to the prediction and differ increasingly with loading rate. However, we remark that other differences between theory and model may alter the behavior. First, inhomogeneity of the simulated fault will arise due to stress fluctuations, slight changes in frictional properties and imperfections in the slip surface due to nonplanarity, wear, or microcracks. Second, the actual frictional behavior of the simulated fault may not be perfectly matched by the specific rate‐and‐state friction that is being assumed in the stability analysis.

In the rate‐and‐state friction framework, the friction coefficient can be expressed as in Ruina (1983):

(4) $$\tau =\sigma_{n}\;\left(\mu_{0}\;+\;a\ln\left(\frac{V}{V_{0}}\right)\;+\;b\ln\left(\frac{V_{0}\theta }{d_{c}}\right)\right),$$

where for the aging law

(5) $$\frac{\mathrm{d\theta}}{\mathrm{dt}}=1-\frac{\mathrm{V\theta}}{d_{c}}$$

and for the slip law

(6) $$\frac{\mathrm{d\theta}}{\mathrm{dt}}=-\frac{\mathrm{V\theta}}{d_{c}}\ln\left(\frac{\mathrm{V\theta}}{d_{c}}\right),$$

where *σ*_*n*_ is the normal stress; *a* and *b* are the rate and state constitutive parameters, respectively; *V* is the slip rate; *μ*_0_ is the reference friction coefficient at the reference slip rate *V*_0_; and *d*_*c*_ is a characteristic slip.

Although no simple analytical solution exists for these laws, the expected critical nucleation length at which a slipping zone becomes unstable has been discussed by several authors (Ampuero & Rubin, 2008; Dieterich, 1992; Fang et al., 2010; Rice, 1993; Ruina, 1983; Rubin & Ampuero, 2005; Uenishi & Rice, 2003). Because of the nontrivial evolution of the coupled parameters *θ* and *V* along a fault obeying a rate‐and state law, the nucleation history and therefore the critical nucleation length can be significantly different depending on which initial and loading conditions are used in numerical models (Fang et al., 2010; Rubin & Ampuero, 2005). Using a simple spring‐slider model and linear stability analysis, Ruina (1983) and Rice (1993) have shown that the critical stiffness of a patch was given by *k*_*c*_=(*b* − *a*)*σ*_*n*_/*d*_*c*_, giving a critical nucleation length *L*_*b* − *a*_=*G* ^∗^ *d*_*c*_/((*b* − *a*)*σ*_*n*_),*G* ^∗^ being the effective shear modulus for in‐plane stress, *G* ^∗^=*G*/(1 − *ν*). Rubin and Ampuero (2005) also examined in detail the nucleation of rate and state faults and showed that the variable Ω=*V* *θ*/*d*_*c*_ played a crucial role in the process. They found that depending on the value of Ω at the time of nucleation, on the ratio a/b, and on the state variable evolution law chosen (slip or aging law), different expression for the nucleation length could be expected (Ampuero & Rubin, 2008; Rubin & Ampuero, 2005). In particular, when Ω≈1, close to steady state, the nucleation length reaches an upper bound $L_{\infty }=G^{*}d_{c}/\pi \times (b/(\sigma_{n}{\left(b-a\right)}^{2}))$. The same scaling in *b*/(*b* − *a*)^2^ can be found in the critical length derived by Andrews (1976) using a slip‐weakening law and an energy criterion *L*_*Andrews*_=2*G* ^∗^ *D*_*c*_/*π* × (*τ*_*p*_−*τ*_*f*_)/(*τ*_0_−*τ*_*f*_)^2^. In the case where the nucleation process is fast enough (e.g., considering a fault that has not recently rupture and with high slip rates), Ω≫1 and does not have the time to decrease inside the slipping area unlike for a slow nucleation process. In this case the nucleation zone shrinks to a minimal value of *L*_*b*_=*G* ^∗^ *d*_*c*_/(*b* *σ*_*n*_). Uenishi and Rice (2003) and Dieterich (1992) also showed that in the case Ω≫1 the rate‐and‐state law can be approximated as a slip‐weakening law, and the critical nucleation length scales as *b* ^−1^. It has also been remarked that low ratios of a/b favor a nucleation patch of size *L*_*b*_ while larger ratios of a/b favor the expanding crack case growing up to a critical length $L_{\infty }$ (Rubin & Ampuero, 2005) unless Ω is very large at the time of nucleation. Finally, by comparing the two evolution laws, Ampuero and Rubin (2008) found that when Ω≫1 they gave similar results, while when Ω≈1 the slip law produced unidirectional rupture propagation only.

In order to compare the experimentally determined nucleation lengths to the theoretical estimates, we use the values from Kaneko et al. (2016), who modeled similar experiments (Latour et al., 2013) that were run with a loading rate of $\dot{\tau }$ = 0.36 MPa/s. The values are summarized in Table 1. However, it is important to point out that in Latour et al. (2013), both shear and normal stresses were time dependent due to the oblique fault in the experimental setup. Also, we use the aging law to estimate the nucleation length and to compare it to *L*_*b*_ and $L_{\infty }$ derived by Rubin and Ampuero (2005). Those values do not necessarily hold for the slip law, and it is not clear which law would be the most representative of the experiments in this study.

**Table 1:** Parameters Used to Estimate the Nucleation Lengths L_b_,L_b − a_ and $L_{\infty }$

Parameter	Value	Unit
*μ*	0.96	GPa
*ν*	0.35	
*d*_*c*_	2 × 10^−7^	m
*σ*_*n*_	4.7	MPa
a	0.01	
b	0.0144	

*Note*. *ν* is the Poisson's coefficient.

Using those parameters, we obtain *L*_*b*_=0.44 cm, *L*_*b* − *a*_=1.43 cm, and $L_{\infty }=1.49$ cm. The measured values *L*_*sc*_ (Figure 4a) are comprised between 0.25 and 1.4 cm, close to the predicted range bounded by *L*_*b*_ and $L_{\infty }$. We do not know what would happen for a larger range of experimental loading rates values; however, we can infer that the values taken by *L*_*sc*_ would tend asymptotically toward the bounds *L*_*b*_ and $L_{\infty }$ for $\dot{\tau }\;\leq$ 6 MPa/s and $\dot{\tau }\;\geq$ 0.01 MPa/s, respectively. Using the energy criterion of Andrews (1976), along with our averaged values of $\bar{\tau_{0}}=1.58$ MPa, $\bar{\tau_{p}}=1.84$ MPa , $\bar{\tau_{f}}=1.05$ MPa, and $\bar{D_{c}}=14\;\mu$m from the experimentally determined friction law (Figure 6e), we obtain *L*_*Andrews*_=3.7 cm, which is slightly more than twice the maximum value $L_{\infty }$. An important result of this study is that we are able to obtain nucleation lengths ranging from the minimum to the maximum values predicted by the rate‐and‐state laws only by varying the loading rate. Even though this parameter is often neglected in theoretical studies to obtain analytical solutions of rate‐and‐state laws, $\dot{\tau }$ seems to have a great influence on the path taken by the coupled parameters *θ* and *V*, which ultimately control the nucleation length. In fact, the loading rate itself may not be the determining factor, but rather the greater acceleration resulting from the imposition of a high loading rate starting from close to zero velocity, thus forcing the system away from the steady state. In particular, if inside the nucleation patch Ω becomes ≫1 due the sudden increase of velocity *V*, and if the state variable *θ* does not have the time to evolve during the nucleation phase due to a high loading rate, Ω will remain ≫1 by the time of instability, in which case a small nucleation length close to *L*_*b*_ is to be expected.

**Figure D5:**
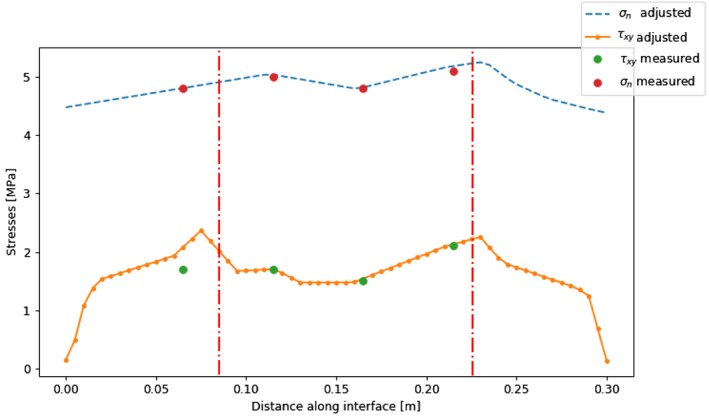
Manually adjusted tractions at the interface to qualitatively fit the fringes.

In addition to the dependence of *L*_*c*_ on $\dot{\tau }$, we also observe a dependence of the nucleation position (see Figure 5). Indeed, while the ruptures nucleate more or less randomly along the interface at low $\dot{\tau }$, as we exceed a value of $\dot{\tau }\;\approx$ 0.3 MPa/s, the rupture initiates systematically within two localized patches positioned at 8.5 and 22.5 cm along the interface. By using a finite element model to match the observed fringes, we show that they seem to correspond to zones of high coulomb stresses, where the nucleation is thus likely to initiate (see Figure D5). Previous studies already showed that the initial stress distribution (N. Kato & Hirasawa, 1996) and frictional parameters *a* and *b* (Kawamura & Chen, 2017; Ray & Viesca, 2017) would influence the rupture nucleation, but the role of the loading rate was not clear. In more recent studies, Xu et al. (2017) who observed a similar negative dependence of Lc with $\dot{\tau }$ showed that the spatial distribution of the nucleation zones of stick‐slip events were also influenced by $\dot{\tau }$. However, in their case the effect was the opposite compared to our experiments: The nucleations occurred all within the same patch for loading rates of 0.01 and 0.1 mm/s but started to be located randomly at 1 mm/s. This contrast between the experiments presented here and the results of Xu et al. (2017) could be explained by a different frictional evolution of the precut surfaces between experiments using granite samples (possible frictional melt during weakening phase, which would solidify during the healing process) or polycarbonate in our study (no melt during dynamic ruptures, but rather elasto‐plastic deformations). In addition, between each event we reset the plates to their initial positions and wait approximately 20 s in order for the interface to heal while in the case of Xu et al. (2017), each experiment at a given loading rate comprises a continuous series of stick‐slip instabilities.

**Figure 7:**
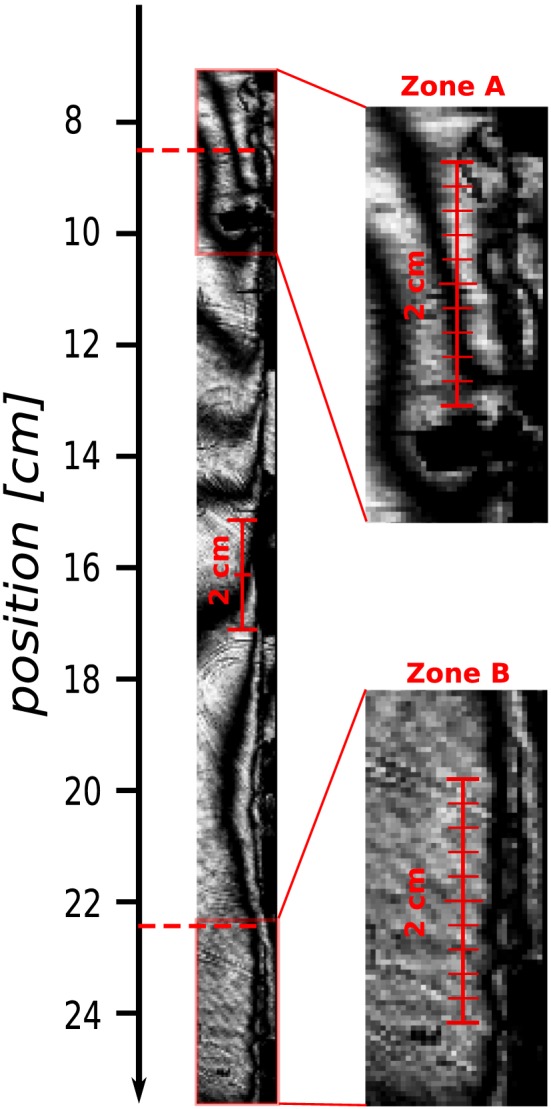
Zoom‐in of the photoelastic fringes located around the high‐stress areas (zones A and B), showing short wavelength variations of stress between 4 and 8 mm, compared to the smoother distribution at the center of the frame.

Although we do not have a clear explanation why some rupture would nucleate in zones of lower coulomb stress, it is very likely that there exist smaller stress heterogeneities that we are not able to resolve with the sparse strain gage measurements or on the isochromatic fringes, and which may vary from one experiment to another influencing the nucleation position; the nucleation may start at some sites where locally high coulomb stress is not resolved by our measurements. The discussion in Appendix D gives only a general idea of the stress distribution along the interface but does not enable to resolve very small stress variations or to compare quantitatively the initial stresses of different experiments. We also note that the wavelength of the stress heterogeneity along the interface may play a role in the nucleation localization. Indeed, in the preferred nucleation zones A and B (see Figure 7), a cluster of heterogeneities of smaller wavelength compared to the rest of the interface (4 to 8 mm) is visible in the photoelastic fringes. One hypothesis is that fast stressing would favor the instability closer to small‐scale inhomogeneities, while gradual stressing favors the development of preslip on larger, homogeneous patches. This could be the case if the stress redistribution during the rupture preparation phase followed a diffusive process, because the diffusion time is proportional to the square root of the inhomogeneity wavelength. Under slow loading the small wavelength heterogeneities would have time to disappear, favoring the development of larger and longer‐lasting stress variations. In this study, we have no direct experimental evidence of such process other than the presence of small‐scale heterogeneity as illustrated in Figure 7, and at time of failure, the level of small‐scale heterogeneity appears to be the same in either fast‐ or slow‐loading conditions. However, as mentioned earlier, it is difficult to make an accurate quantitative analysis based on the photoelastic images alone, and the stress is measured only at the four sites, which were instrumented with strain gages. Also, we can visualize only the main rupture at a late nucleation stage: Some rate‐dependent slip may occur during the previous seconds of loading, which are not captured. Numerical experiments using heterogeneous a and b values and/or heterogeneous initial stress distribution along with varying loading conditions (different loading rates and hold times) would be needed to better understand the influence of the loading rate on the nucleation position.

**Figure 8:**
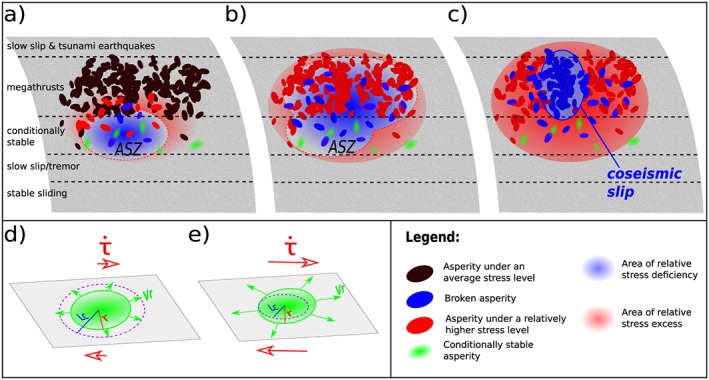
Cartoon illustrating how the loading rate may locally increase in a slab close to rupture (a,b,c), and how such loading rate increase can induce seismic rupture of conditionally stable asperities (d,e). a) An apparent slipping zone (ASZ) expands from the conditionally stable part, slowly releasing stress in the blue area and increasing it outside in the red area. Blue asperities are failing; red asperities are locked and accumulating stress; black asperities (located further away) not yet significantly affected the stress variations; green asperities are conditionally stable. The conditionally stable asperities (green) do not fail seismically at this stage because their radii r are smaller than L_c_. (b) The ASZ slowly expands, inducing an increase in both load and loading rate in the surrounding area. This activates seismic failure of either previously locked asperities, or of previously aseismic, conditionally stable asperities (green) due to the shrinking of L_c_ below their radius r. (c) Final stage of nucleation for a large earthquake. A dense cluster of asperities fail jointly (cascade or preslip model), further accelerating load around the slip area and finally triggering a large seismic rupture. (d) When the loading rate is relatively low (stage a), the conditionally stable asperities slip aseismically as L_c_. is larger than the asperity radius r. When the loading rate increases (stages c and d), the previously aseismic asperities might start to fail seismically if L_c_ becomes smaller than r.

To understand what those experimental results can mean for real earthquakes, it is important to have a global picture of how the interface between plates behaves. From recent observations where preslip was detected before an earthquake by inverting GPS signals (Ruiz et al., 2014, 2017), a large Apparent Slipping Zone, which we will refer to as ASZ, appears to be activated around the future epicenter of the earthquake. In order to understand why this ASZ (interpreted as the preseismic slip) is larger than the coseismic slip, it is important to imagine this ASZ as a highly heterogeneous patch actually composed of smaller locked (or rate‐weakening) and creeping (or rate‐strengthening) patches (see Figure 8a), as suggested by Socquet et al. (2017). Those patches are progressively activated as the ASZ expands (Figure 8b), some asperities (intended as either conditionally stable or unstable velocity‐weakening patches) might fail (foreshocks), releasing stress locally and in the surrounding creeping material, while others might remain locked and build‐up stress. It has been observed that this apparent homogeneous ASZ is not only discontinuous in space but also in time (Frank et al., 2017). The remaining locked asperities inside the ASZ will still be progressively loaded as it expands, and if a large cluster of them breaks (see Figure 8c), this would be the main shock of an earthquake and possibly a foreshock of a next one.

In our case, it is hard to tell if the *L*_*sc*_ measured from the experiments (a few centimeters) can be extrapolated to infer the size of the ASZ, which can reach a radius of several tens of kilometers (Ruiz et al., 2014). Indeed, the ASZ is very heterogeneous and experiences much more complexity in the frictional evolution than in the controlled laboratory experiments. But considering that the ASZ may be close to velocity neutral, with an average of (a‐b) close to 0, the estimates *L*_*b* − *a*_ could be infinitely large.

Contrary to the observations reported by Ruiz et al. (2014), the conditions under which the experiments are conducted here do not appear to result in a large creeping patch with locked asperities, but rather in a locked fault with a localized preslip patch that grows into a dynamic rupture, corresponding rather to the observations of Tape et al. (2018) for a M3.7 asperity. We can therefore discuss the possible effects of a shrinking nucleation size under accelerated loading rate in some natural contexts, for example, linking the natural earthquake nucleations described by Ruiz et al. (2014) and Tape et al. (2018) or to explain the appearance of aftershocks following the 2011 M9.0 Tohoku‐oki earthquake, in places where only very few earthquakes had been observed during the last 88 years (Hatakeyama et al., 2017).

A locked asperity on a creeping fault could fail aseismically: If *L*_*c*_ were larger than the size of the asperity, the slip would not accelerate up to seismic velocities needed to radiate waves (Mclaskey & Yamashita, 2017; see Figure 8d). However, if the loading rate in the vicinity of this patch is suddenly increased, *L*_*c*_ could decrease below the asperity dimensions, and the patch could become seismic (see Figure 8e). As noted by Mclaskey and Yamashita (2017) this model is consistent with the observations of Wech and Bartlow (2014) who evidenced a correlation between slow slip rate and the number of tremors in Cascadia, considering that a subduction interface can be composed of a large population of those patches oscillating between stable and unstable behavior. As the ASZ undergoes accelerated creep, the local loading rate on the smaller locked asperities increases, and this could trigger their seismic rupture during the preseismic interval. In the postseismic phase, as long as the accelerated creep continues, the small asperities may still undergo seismic rupture as observed in the seismic cycle (Yao et al., 2017), and aftershocks could appear in conditionally stable areas following the increase of loading rate as discussed by Hatakeyama et al. (2017).

## 5 Conclusions

We conducted stick‐slip laboratory experiments as an analog to earthquake rupture, using polycarbonate slabs in frictional contact under biaxial load. The setup allows to maintain the fault under constant normal stress (≈5MPa) while the shear stress is increased at an arbitrary rate from a minimum of 0.01 up to 6 MPa/s. Under these conditions, spontaneous dynamic sliding develops under a range of shear stress values, starting from a slow‐growing nucleation region of a few millimeters from which a (mostly bilateral) rupture front departs and accelerates up to either sub‐Rayleigh or intersonic velocities, in agreement with previous observations Latour et al. (2013), Nielsen et al. (2010), Ohnaka and Kuwahara (1990), and Schubnel et al. (2011), although experimental conditions and materials differ. The rupture nucleation and dynamic propagation were monitored by both high‐speed photography (revealing the photoelastic fringe patterns related to the maximum shear stress) and four strain‐gage rosettes (3 × 4 strain gages, sampling the full in‐plane strain at 4 points along the fault length).

Our main focus here is the nucleation size, its variability, and how it correlates with the loading rate. This is explored in response to numerical results (Kaneko et al., 2016) that indicated that nucleation size may be affected by loading rate. We find indeed that faster loading results in reduced nucleation size, with a minimum of *L*_*c*_≈0.8cm, while maximum nucleation length of *L*_*c*_≈2.4cm is obtained for slow rates.

A first‐hand interpretation can be made assuming that rate‐and‐state friction controls slip in the slow phase of nucleation preceding the dynamic instability. In this case, a slower loading rate would allow to be closer to the steady state, where the dimensionless variable *V* *θ*/*d*_*c*_ is close to 1. Faster loading may imply that the fault is further from the steady state, as *V* increases, but *θ* has no time to evolve, resulting in larger values of *V* *θ*/*d*_*c*_. As pointed out by Rubin and Ampuero (2005), numerical simulations indicate that the two limit sizes *L*_*b*_ and $L_{\infty }$ are selected depending on whether the value of *V* *θ*/*d*_*c*_ is large or small, respectively. The upper and lower bounds for nucleation length observed in our experiments are compatible (within the error bar) with values of $L_{\infty }$ and *L*_*b*_, respectively, derived with rate and state parameters (*a*,*b*,*d*_*c*_) corresponding to our simulated fault. The nucleation length is also compatible, within a factor of 2, with the critical length proposed by Andrews (1976) for earthquake fault slip, equivalent to the Griffith‐Irwin rupture criterion based on energy balance.

In addition to the loading rate dependence of nucleation size, we also observe an effect on the location of the nucleation. Fast loading rates result in systematic nucleation within two individual patches, whereas slow loading results in a random position of nucleation. Short wavelength stress perturbations are visible in the regions of the two individual patches. One hypothesis is that nucleation is favored on shorter wavelength stress concentrations under fast loading; this could occur if stress redistribution along the fault did occur under a diffusive‐type process, where shorter wavelengths dissipate at faster rate. Using the photoelastic stress fringes in conjunction with the stress sampled at the four instrumented points in a trial‐an‐error inverse method, we proposed a model of prerupture state of stress on the experimental fault, compatible with the experimental loading conditions. Although the stress distribution matching the data is highly nonunique, the preferred model shows two peaks in Coulomb stress at sites of the systematic nucleations under fast loading. Surprisingly, the stress concentrations seem to persist even under the low‐rate loading conditions, where nucleation sites are randomly distributed.

We also focused our analysis on the shape of the slip function and the shear stress evolution during rupture, at the 4 points instrumented with strain gages along the fault. We find that the friction drops primarily with slip rather than with slip velocity, although it is clear that a relatively high slip velocity is necessary to achieve weakening. Considering the well‐known flash heating model for frictional weakening (Archard, 1959; Rice, 2006), an inverse velocity dependence of friction expected is based on the temperature rise within the contact asperities. The experimental conditions explored here allow for a very modest temperature rise; therefore, flash heating is not expected, neither is the direct velocity dependence of friction. The weakening distance is approximately 14μm and does not show any systematic variability with loading rate, distance from the nucleation site, or final slip amount. Typical slip pulses last about 100 to 200 μs; no dynamic restrengthening is observed; therefore, slip halt is induced by stopping phases from the rupture front arrest, or by reflection from the sample boundaries, as in typical crack‐like rupture models.

Finally, we discuss the implications of our findings for natural earthquakes. Although the change in scale from our experiments to moderate or large magnitude earthquakes is several orders of magnitude, in principle the scaling of the nucleation problem is such that an adequate choice of parameters allows to produce an arbitrarily large nucleation length (e.g., for values of rate and state parameters (*a* − *b*)→0^−^). Therefore, the experimental observations conducted here, with all due consideration to the possible differences in setting, scale, time interval, geometry, and parameters, can be in principle up‐scaled to make inferences about natural seismicity, where a loading‐rate dependence of the nucleation size may also be expected. For example, we comment that accelerated slip is observed to trigger small‐size seismic ruptures that cluster within fault areas that are normally under aseismic creep. If the the critical nucleation length was normally larger than the size of the asperities responsible for such seismic ruptures, they would be able to fail seismically only if the nucleation length dropped, which may the case under accelerated loading as indicated by the experiments presented here.

## Figures and Tables

**Figure B1:**
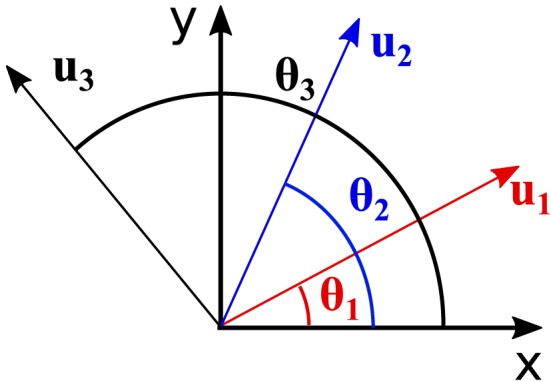
Rotation of strains.

**Figure D1:**
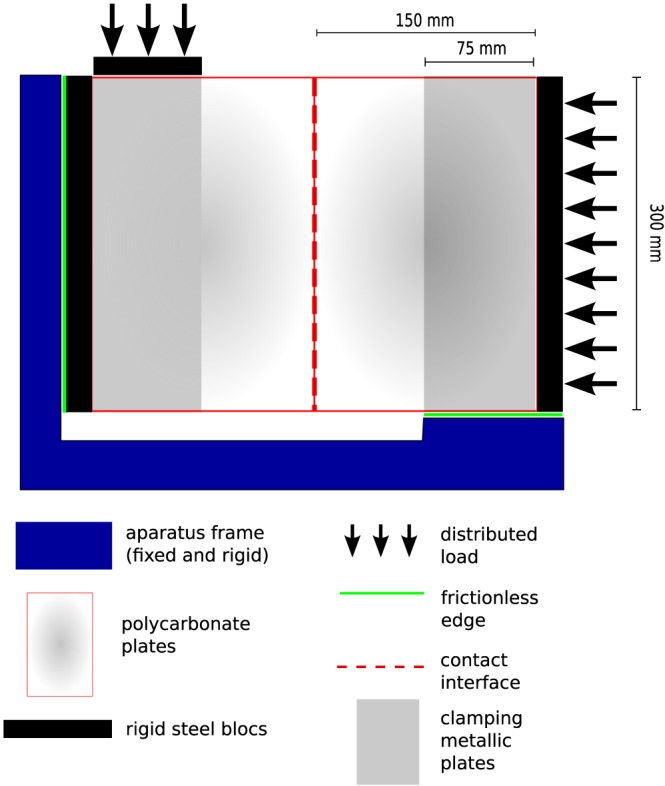
Sketch of the experimental setup and boundary conditions of the model.

**Figure D2:**
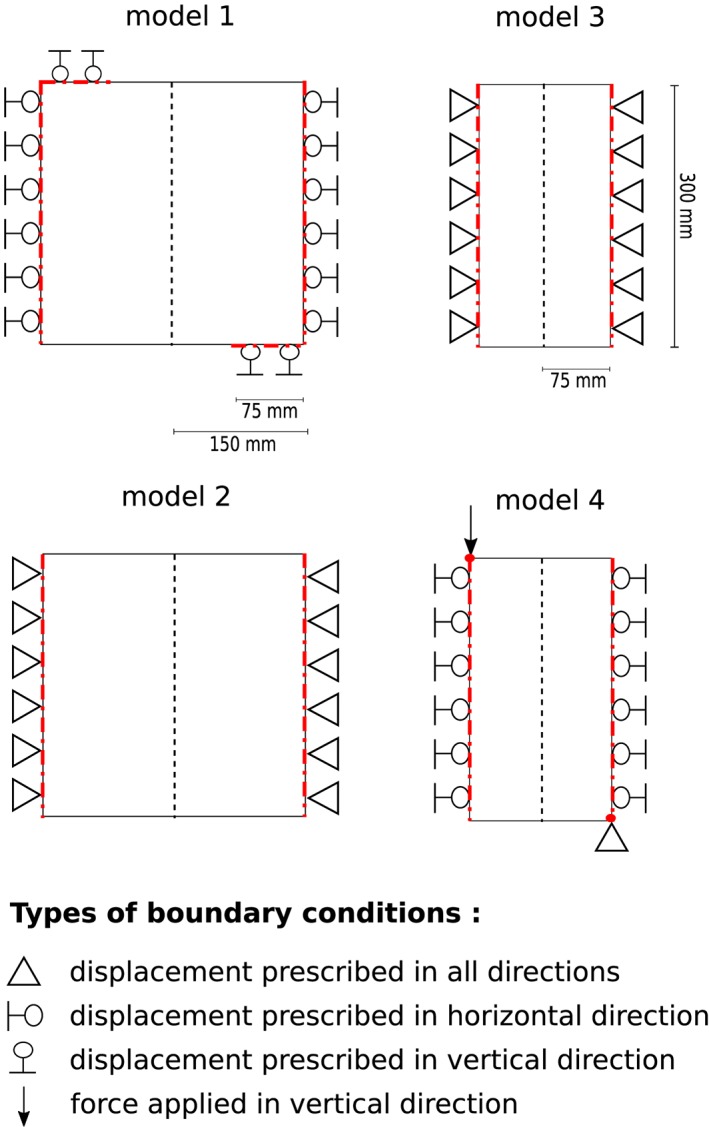
Different types of numerical models used to generate shear stress at the interface (dashed line).

**Figure D3:**
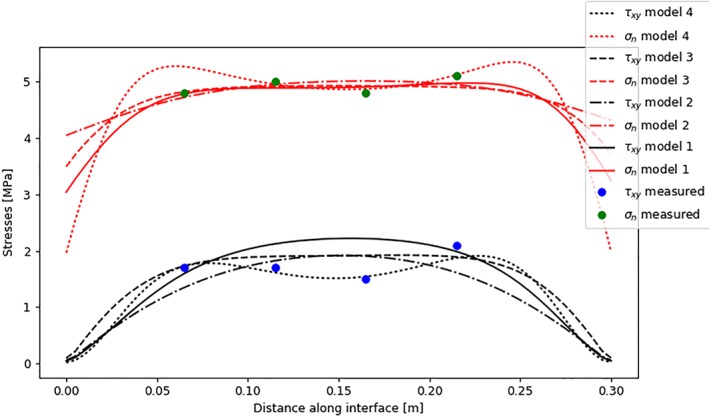
Shape of shear stresses generated at the interface by each of the four loading configuration, and strain gage point measurements of shear stresses.

**Figure D4:**
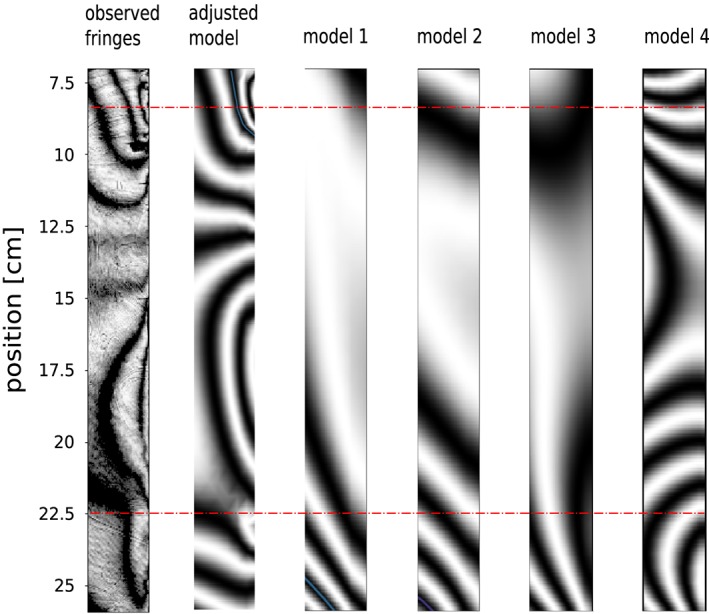
Fringe patterns simulated in the zone corresponding to where the experimental fringes have been filmed.

**Figure D6:**
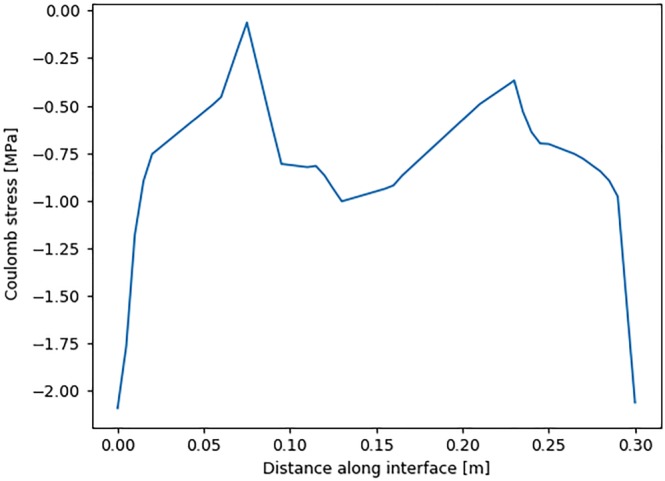
Coulomb stress from the adjusted model using a friction coefficient μ = 0.5. The closer τ_coulomb_ is to 0, the closer to failure this point is.

## Appendix B Stresses Calculation

### B.1

We use strain gages with a three‐wire quarter‐bridge configuration and measure the output voltage *V*_*o**u**t*_(*t*). Adjusting the output voltage *V*_*o**u**t*_=0 when *ε* = 0, then *ε*(*t*) can be obtained by the relation *ε* =− 4*V*_*o**u**t*_(*t*)/[*G*_*f*_(*V*_*i**n*_+2*V*_*o**u**t*_(*t*))], where *V*_*i**n*_ is the bridge excitation voltage and *G*_*f*_ is the gage factor.

The rosette strain gages enable to measure the 2D strain tensor and we assume plane stress conditions. The 3 stacked gage grids (see Figure 1.2) enable to measure the strains *ε*_1_,*ε*_2_, and *ε*_3_ in three different orientations *θ*_1_,*θ*_2_, and *θ*_3_, along axes *u*_1_,*u*_2_, and *u*_3_, respectively (Figure B1).

If x is the axis aligned with the interface, we can invert the parallel, perpendicular, and shear components of strain ε_xx_,ε_yy_, and γ_xy_ using

$$\varepsilon_{i=1,2,3}=\varepsilon_{\mathrm{xx}}{\left(\cos\theta_{i}\right)}^{2}+\varepsilon_{\mathrm{yy}}{\left(\sin\theta_{i}\right)}^{2}+\gamma_{\mathrm{xy}}\sin\theta_{i}\cos\theta_{i},$$

which in our case (θ_1_ = 45° , θ_2_ = 90° , θ_3_ = 135° ) reduces to

$$\varepsilon_{\mathrm{xx}}=\varepsilon_{3}+\varepsilon_{1}-\varepsilon_{2},$$

$$\varepsilon_{\mathrm{yy}}=\varepsilon_{2},$$

$$\gamma_{\mathrm{xy}}=\varepsilon_{1}-\varepsilon_{3}.$$

The stresses can then be retrieved using Hooke's law:

$$\sigma_{\mathrm{xx}}=\frac{E}{1-\nu^{2}}(\varepsilon_{\mathrm{xx}}+\nu \varepsilon_{\mathrm{yy}}),$$

$$\sigma_{\mathrm{yy}}=\frac{E}{1-\nu^{2}}(\varepsilon_{\mathrm{yy}}+\nu \varepsilon_{\mathrm{xx}}),$$

$$\sigma_{\mathrm{xy}}=G\gamma_{\mathrm{xy}}=\frac{E}{2(1-\nu )}\gamma_{\mathrm{xy}},$$

where E is the Young's modulus.

## Appendix C Slip Calculation for the Friction Law

### C.1

In order to obtain the relative slip to plot the friction laws, we use the same method that in Svetlizky and Fineberg (2014), and assuming that the rupture front is propagating at approximately constant velocity V_r_ (we can check using the high‐speed camera), we can obtain the local slip velocity V(x,t) (as twice the particle velocity on one side of the fault), using the measurement of fault‐parallel strain:

$$V(x,t)=2\;\frac{\mathrm{\partial u}(x,t)}{\mathrm{\partial t}}=2\;\frac{\mathrm{\partial u}(x-V_{r}t)}{\mathrm{\partial t}}=2\;\frac{\mathrm{\partial u}}{\mathrm{\partial x}}\frac{\mathrm{\partial x}}{\mathrm{\partial t}}=-2\;V_{r}\varepsilon_{\mathrm{xx}}(x,t),$$

where u is the particle displacement in the x direction (parallel to the fault). Now defining t = t_0_ as the time just before a rupture front passes at a position of the strain gage at x, and $\varepsilon_{\mathrm{xx}}^{0}=\varepsilon_{\mathrm{xx}}(t_{0})$, we obtain fault slip U as

$$U(x,t)=\int_{t_{0}}^{t}2\frac{\mathrm{\partial u}(x,t^{\prime })}{\partial t^{\prime }}dt^{\prime }=-2\;V_{r}\int_{t_{0}}^{t}\left(\varepsilon_{\mathrm{xx}}(x,t^{\prime })-\varepsilon_{\mathrm{xx}}^{0}\right)dt^{\prime }.$$

## Appendix D Initial Stress Distribution of Dynamic Ruptures Inferred From Photelastic Fringe Patterns Coupled With Finite Element Models

### D.1

This section aims at discussing what is the stress distribution along the interface between the two polycarbonate plates, just before a rupture, using the photoelastic fringe patterns and finite element method (FEM).

As described in section 2, the setup is composed of two polycarbonate plates 15 cm wide by 30 cm long, and 1 cm thick (see Figure D1). They are clamped by two metallic frames overlapping them over a width of 7.5 cm, on each side of the setup. Then two pistons are applying a normal force by pushing uniformly on the right edge, 30 cm long. Once under normal load, a fault parallel shear stress is applied by pressing the left clamping frame downwards. Although the setup seems simple, further complexity arises from the fact that the plate might actually not be clamped uniformly within the width of the metallic frame. And this could generate different loading conditions, therefore different stresses at the interface.

In order to discuss which loading configuration is the best suited to represent the experiments, we perform finite element simulations using different boundary conditions as described in Figure D2. Note that if the plates were perfectly maintained within the metallic clamp, we should only model two elastic domains 7.5 cm wide and 30 cm long (models 3 or 4 Figure D2). But if they are allowed to move inside the clamps, it might resemble rather models 1 and 2 of Figure D2.

Using the loading configurations illustrated in Figure D2, we first adjust the magnitude of the forces or displacements to match roughly the strain gage measurements of shear and normal stresses to the stresses calculated in the middle of the finite element method models (dashed lines, Figure D2). At this stage we observe that for models 1 and 2 (30 by 30 cm), the shear distributions along the interface tend toward parabolic‐like shapes and as we shorten the models (models 3 and 4), we start to see corners appearing at the edges (Figure D3). In all the cases the shear stress goes down to 0 at the edges, which is to be expected at free boundaries. The normal stresses are more homogeneous and more or less similar between the different simulations except for model 4 where we apply point loads. All the normal stress distributions present corners where the stress drops roughly 7 cm away from the edges. Model 4 is the one that seems to match best the shear stress distribution, while the normal stress distribution is better represented by the other models.

In order to compare the models we also simulate the isochromatics that the shear and normal stress values measured at the interface would generate if we applied them as tractions along the 30‐cm‐long edge of one single plate, taking a fringe value *f*_*σ*_= 4,000 N/m. The opposite edge is left with the same boundary type as it is in Figure D2. We compare the patterns of the four models to each other, to a fifth model where shear and normal stresses were manually adjusted to match the isochromatics (see Figure D5), and to a typical snapshot of fringe pattern before a rupture (Figure D4). The boundary conditions of the fifth model are like the model 3 on the right edge (fixed in all directions). Models 1 and 2 seem far from matching the isochromatics while models 3 and 4 seem to be a better starting point. It is thus important to consider the plates as shortened to half their original width, as they are clamped between the metallic frames. Then starting from model 3 and by manually adjusting the tractions at the left edge, we manage to obtain a fringe pattern qualitatively similar to the one observed (adjusted model and observed fringes in Figure D4). This requires two peaks of shear stress at approximately 8 and 23 cm along the interface, while the normal stress is more homogeneous, and only dropping slightly on the edges according the model of Figure D5.

The adjusted model is consistent with the observed nucleation positions at 8.5 and 22.5 cm as the coulomb stress of the model *τ*_*c**o**u**l**o**m**b*_=*τ*_*x**y*_−*μ* *σ*_*n*_ is close to zero at those locations (see Figure D6). The rupture is therefore more prone to start from those two locations.
